# Documenting Greek Indigenous Germplasm of Cornelian Cherry (*Cornus mas* L.) for Sustainable Utilization: Molecular Authentication, Asexual Propagation, and Phytochemical Evaluation

**DOI:** 10.3390/plants11101345

**Published:** 2022-05-19

**Authors:** Eleftherios Karapatzak, Nikos Krigas, Ioannis Ganopoulos, Katerina Papanastasi, Dimitris Kyrkas, Paraskevi Yfanti, Nikos Nikisianis, Antonis Karydas, Ioannis Manthos, Ioanna S. Kosma, Anastasia V. Badeka, Dimitrios Fotakis, Eleni Maloupa, Giorgos Patakioutas

**Affiliations:** 1 Institute of Plant Breeding and Genetic Resources, Hellenic Agricultural Organization Dimitra, 57001 Thessaloniki, Greece; ekarapatzak@gmail.com (E.K.); giannis.ganopoulos@gmail.com (I.G.); papanastasi@bbgk.gr (K.P.); euripidis_1999@hotmail.com (A.K.); manthosjo@yahoo.gr (I.M.); maloupa@bbgk.gr (E.M.); 2 Department of Agriculture, University of Ioannina, 47100 Arta, Greece; dkyrkas@uoi.gr (D.K.); pyfanti@uoi.gr (P.Y.); 3 Systada General Partnership, 55133 Thessaloniki, Greece; nnikisia@gmail.com; 4 Laboratory of Food Chemistry, Department of Chemistry, University of Ioannina, 45110 Ioannina, Greece; i.kosma@uoi.gr (I.S.K.); abadeka@uoi.gr (A.V.B.); 5 Forest Research Institute, Hellenic Agricultural Organization Dimitra, 57006 Thessaloniki, Greece; fotakis@fri.gr

**Keywords:** neglected and underutilized plants, phytogenetic resources, DNA barcoding, forest berries, protocols, nutraceutical potential, genotype selection, multifaceted evaluation

## Abstract

Wild-growing Cornelian cherries (*Cornus mas* L., Cornaceae) are well-known native fruits in Greece since ancient times that are still consumed locally nowadays. Modern research has highlighted the value of Cornelian cherries as functional food with exceptional health benefits on account of the fruits’ biochemical profile. However, apart from local consumption directly from wild growing individuals, Greek native *C. mas* populations have not yet been investigated or sustainably utilized. A multifaceted evaluation was conducted herein including authorized collection-documentation, taxonomic identification, and molecular authentication (DNA barcoding), asexual propagation via cuttings and phytochemical evaluation (multiple antioxidant profiling) of neglected and underutilized Greek native *C. mas* germplasm sources. Successive botanical expeditions resulted in the collection of 18 samples of genotypes from distant *C. mas* populations across different natural habitats in Greece, most of which were DNA fingerprinted for the first time. Asexual propagation trials revealed high variability in rooting frequencies among Greek genotypes with low (<25%), average (25–50%), and adequate propagation potential (>50%) using external indole-3-butyric acid (IBA) hormone application on soft- or hard-wood cuttings. The comparative phytochemical evaluation of the studied Greek genotypes showed significant potential in terms of antioxidant activity (>80% radical scavenging activity in 13 genotypes), but with variable phenolic content (47.58–355.46 mg GAE/100 g), flavonoid content (0.15–0.86 mg CE/100 g), and vitamin C content (1–59 mg AAE/100 g). The collected material is currently maintained under ex situ conservation for long-term monitoring coupled with ongoing pilot cultivation trials. The pivotal data create for the first time a framework for the sustainable utilization of Greek native C. mas germplasm as a superfood with significant agronomic potential.

## 1. Introduction

Apart from widely used crop varieties, phytogenetic resources also include wild-growing plant species which are often neglected or underutilized, especially in biodiversity-rich areas [1]. Such germplasm resources involve a wide variety of plant species, including small fruit trees of potentially high alimentary value, relating to nutritional health benefits, but also pharmaceutical value [2,3,4,5,6]. Local native fruit species can be an important source of natural food products rich in antioxidants such as polyphenols with known importance for human health [7,8,9]. The natural heritage of Greece and its rich biodiversity at all levels offer new possibilities for evaluating and utilizing valuable wild germplasm such as small stone fruits and berries with high nutritional and medicinal (in one word nutraceutical) value [6,10]. Prior to domestication and sustainable exploitation of wild germplasm resources of interest, multifaceted research approaches are required in terms of taxonomic validation and molecular authentication of wild plant material, agronomical assessment propagation-wise, and comprehensive phytochemical evaluation aiming at the selection of promising genotypes with high potential for further applied research and future breeding. To this end, Greek native *Rosa canina* and *Sambucus nigra* germplasms constitute recently developed example cases [6,10]. 

Cornelian cherry (*Cornus mas* L., Cornaceae) is a rather common and wild-growing deciduous shrub or occasionally a small tree 2–4(–6) m tall in scrub and woodlands of central and southeastern Europe and western Asia, often occurring in ravines or near streams (areas with sufficient rainfall) from low (100–200 m) to intermediate and higher altitudes (800 m, occasionally up to 1700 m) [11]. In Greece, Cornelian cherry is a traditionally well-known native fruit tree that has been consumed locally for centuries and it has been known since ancient times, i.e., the Trojan horse in Homeric times was believed to be a *C. mas* woodcraft [12]. The trees bloom by the end of winter and in early spring and set fruits (ellipsoid to broadly cylindrical drupes 12–15 mm long, becoming red and cherry-like) in late summer through fall. Cornelian cherry fruits (both wild-growing and cultivated) and their food derivatives (e.g., jams, jellies, juice) contain antioxidants, phenolic compounds, iron, potassium, vitamin C, flavonoids, and many other substances of high nutritional value [13,14,15,16,17]. In addition, *C. mas* fruits to date have validated pharmaceutical value in terms of free radical scavenging potential among others, resulting in several health benefits including antimicrobial, antidiabetic, anti-inflammatory, anticancer, and cardioprotective activity; these assets render *C. mas* fruits as functional food products and as ‘superfood’ with intensifying relevant research over recent years [18,19,20]. Apart from the flesh of the fruits, the endocarp of *C. mas* drupes has also been demonstrated to have nutraceutical potential as a source of bioactive compounds with strong antioxidant activity [21]. Sustainable utilization efforts of *C. mas* germplasm have been attempted in eastern European countries with promising results [22,23,24]. In more recent studies in the same area, the phytochemical evaluation of *C. mas* domesticated germplasm (cultivars and/or hybrids) has been conducted [22]. Furthermore, *C. mas* germplasm has been characterized in terms of the nutritional value of the fruits across a wider spectrum of its distribution range [25,26,27,28] and the results showed the nutritional superiority of wild-growing germplasm [29]. However, in these studies no assessment was included regarding the native *C. mas* wild-growing genotypes occurring in Greece. 

To support classical taxonomic identification and to enable the establishment of a distinct genetic identity, molecular authentication can be performed through novel DNA barcoding and bioinformatic analysis methods in selected plant materials collected from natural habitats [30]. DNA barcoding as a tool for molecular authentication of plant germplasm has been greatly developed and to date is being widely used for many medicinal plant species and phytogenetic resources [6,10,31,32,33,34,35]. There are also reports of DNA barcodes being successfully used in identification of ancient DNA (aDNA) of *C. mas* from fossils [36]. However, such molecular tools are still underused in authenticating native *C. mas* germplasm of different regions. 

To date, wild-type populations of Cornelian cherry (*C. mas*) from several parts of the world have been successfully propagated via cuttings in the past with the use of external hormone application on hardwood and soft wood cuttings taken from mature individuals growing in the wild [37,38]. A popular hormonal substance that has been used in the past is indole-3-butyric acid (IBA) [39,40], frequently achieving high rooting rates. 

In this framework, the study herein was focused on the collection and multifaceted documentation of *C. mas* plant material from geographically separated Greek populations (different genotypes) with the aim to provide: (1) molecular authentication of the population samples via DNA barcoding; (2) asexual propagation protocols for the collected genotypes via propagation trials using cuttings (dormant hardwood twigs, fresh softwood plant parts) facilitating the ex situ conservation and evaluation of the collected germplasm; and (3) comparative phytochemical evaluation of their fruits in terms of nutraceutical properties (total phenolic content, antioxidant activity, total flavonoids, and vitamin C content) to assess their potential as germplasm sources for artificial selection of genotypes and future breeding efforts. The overall work provided first-time insight into Greek native *C. mas* (Cornelian cherry) germplasm and these efforts are aimed at paving the way for its sustainable utilization as a superfood with significant agronomic potential. 

## 2. Results

### 2.1. Molecular Authentication of Greek Native Cornus mas Genotypes

**Figure 1:**
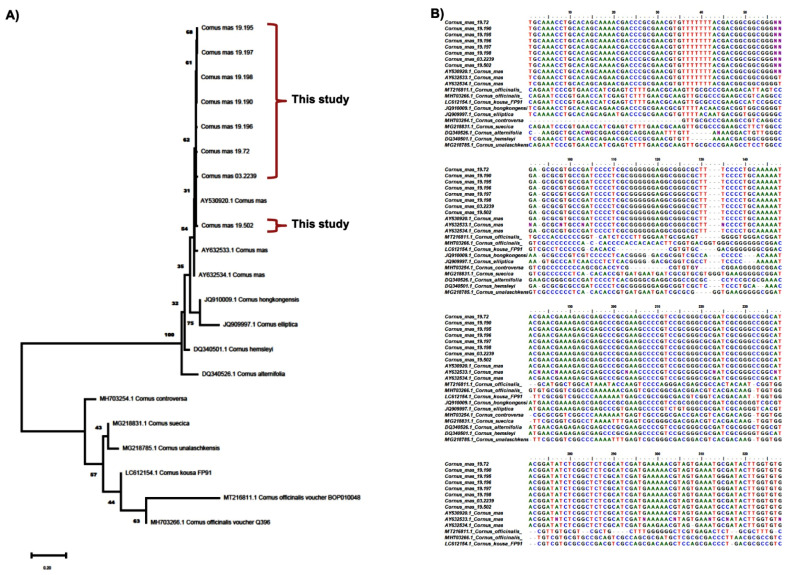
(**A**) Phylogenetic tree on the basis of ITS2 regions regarding the Greek native Cornus mas genotypes in contrast with other C. mas and Cornus spp. genotypes of different origin retrieved from NCBI. (**B**) Overview of the genotypes analyzed in this study with multiple sequence alignment of their ITS2 barcode region. Results from neighbor-joining (NJ) bootstrap analyses with 1000 replicates were used to assess the strength of the nodes. The node numbers indicated the bootstrap value of NJ. The distinct genotypes of this study are highlighted with blue.

BLAST1 and distance-based methods were selected to validate the authentication efficiency of ITS2 for the selected Greek native *Cornus mas* genotypes. The ITS2 barcode with BLAST1 showed a high competence (97% and 100%) in sample identification at species and genus levels, respectively. The ITS2 barcode with the DISTANCE method showed almost similar identification efficiency (98%) at the species level. Thus, barcode ITS2 using the NJ tree method was proved able to distinguish Greek native *C. mas* genotypes from other genotypes of different origin, and similarly, from other *Cornus* spp. not present in Greece (Figure 1A). The neighbor-joining (NJ) phylogenetic tree obtained from DNA barcoding application with the ITS2 region discerned two main groups among the samples of *Cornus* spp. used herein (*n* = 21), and clearly classified the Greek native *C. mas* genotypes of this study as distinct sub-group with respect to other samples of *C. mas* of different origins that were sourced from databases (Figure 1A). The nucleotide differences in Greek genotypes are depicted in Figure 1B. The separation of the genotypes in Figure 1A presents similarity to an extent with the geographical separation of the respective habitats since genotypes GR-1-BBGK-19,195, GR-1-BBGK-19,197, GR-1-BBGK-19,198, and GR-1-BBGK-19,190 coupled with genotype GR-1-BBGK-19,196 originate from areas of the same prefecture, whereas genotype GR-1-BBGK-19,72 is both genetically and geographically further apart (Figure 1A). The same holds true for genotype GR-1-BBGK-19,502 which is geographically separated coming from the lowest altitude. The bootstrap values seem to further validate this classification scheme. Despite the fact that evolutionary relationships may also be analyzed through the neighbor-joining tree, the key function applied herein was to repeatedly evaluate bootstrap values as a measure of informed distinction of the Greek native germplasm (see distinct clades). The results of this study outlined the ITS2 gene as an efficient tool (100%) regarding the distinction of the species in concern (*C. mas*) among other *Cornus* spp. (see monophyletic clusters).

### 2.2. Phytochemical Evaluation of the Greek Native Cornelian Cherries

The phytochemical screening of the fruits of Greek native *C. mas* samples revealed a significant level of variability between genotypes in terms of total phenolic content (TPC), vitamin C content, antioxidant activity (AA), and total flavonoids (TF) (Table 1, *p* < 0.05). No significant correlation with altitude was observed for any of the phytochemical parameters measured. Similarly, no significant correlation of the maturity index of the fruits with any of the phytochemical parameters measured was observed (Table 2, *p* < 0.05, *p* < 0.01). As expected, TPC was significantly positively correlated with TF (*p* < 0.01), while total dissolved solids (TDS) were significantly positively correlated with vitamin C content (Table 2, *p* < 0.05). 

Antioxidant activity (AA) among all assessed genotypes ranged from 55.46 %RSA (radical scavenging activity) in GR-1-BBGK-19,638A to 95.94 %RSA in GR-1-BBGK-19,844 with most genotypes, however, presenting AA above 80% (Table 1, *p* < 0.05). TPC ranged from 29.93 mg GAE/100 g in GR-1-BBGK-19,638A to 355.46 mg GAE/100 g in GR-1-BBGK-19,669. Vitamin C content ranged from 0.95 mg AAE/100 g in GR-1-BBGK-19,847 to 58.97 mg AAE/100 g in GR-1-BBGK-19,638B. Similarly, TF ranged from 0.11 mg CE/100 g in GR-1-BBGK-19,847 to 0.86 mg CE/100 g in GR-1-BBGK-19,669. Most of the genotypes showed very high AA (>80 %RSA) except genotypes GR-1-BBGK-19,638A, GR-1-BBGK-19,753, GR-1-BBGK-19,847, and GR-1-BBGK-19,848 (*p* < 0.05). The same four genotypes also presented the lowest TPC, as expected (Table 1, *p* < 0.05). Very high values of TPC were found in three cases, namely 304.73, 337.14, and 355.46 mg GAE/100 g in genotypes GR-1-BBGK-19,190A, GR-1-BBGK-19,638B, and GR-1-BBGK-19,669, respectively. Concerning vitamin C content, a messier picture emerged with four genotypes presenting very low values of <1.5 mg AAE/100 g including GR-1-BBGK-19,753 and GR-1-BBGK-19,847, similar to AA and TPC results but also genotypes GR-1-BBGK-19,72 and GR-1-BBGK-19,590 which performed better in TPC and AA. All other genotypes showed vitamin C content values from 10 up to 30 times higher than 1.5 mg AAE/100 g (Table 1, *p* < 0.05). Concerning TF, genotypes GR-1-BBGK-19,847 and GR-1-BBGK-19,848 along with GR-1-BBGK-19,195 were among the poorest with 0.11, 0.17, and 0.15 mg CE/100 g, respectively (Table 1, *p* < 0.05).

In addition to the above results, significant year-to-year variations were observed in genotypes that were measured for two consecutive years, especially in TPC. Particularly, genotype GR-1-BBGK-19,190 showed a drop in TPC from 304.73 mg GAE/100 g in 2019 to 49.29 mg GAE/100 g in 2020 coupled with a drop in TF from 0.73 to 0.22 mg CE/100 g (Table 1, *p* < 0.05). On the contrary, genotypes GR-1-BBGK-19,633 and GR-1-BBGK-19,638 showed an increase in TPC between 2019 and 2020 from 52.31 to 195.2 mg GAE/100 g and from 29.93 to 337.14 mg GAE/100 g, respectively, with the latter showing also a significant increase in AA (Table 1, *p* < 0.05). 

### 2.3. Propagation of Greek Native Cornus mas Genotypes with Cuttings

**Figure 2:**
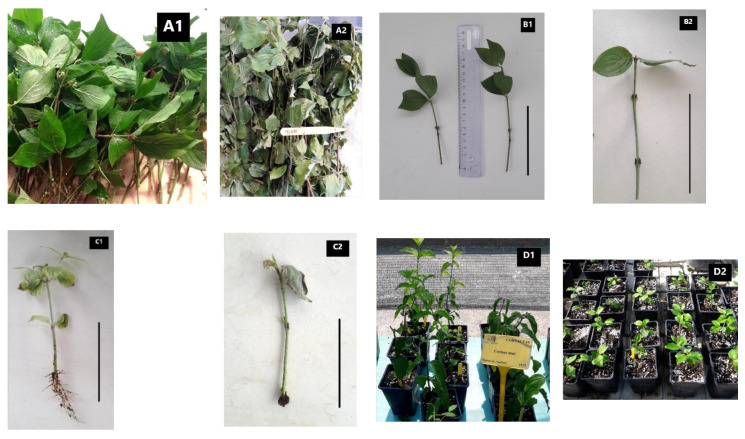
Softwood propagation material collected from representative wild-growing Greek native *C. mas* genotypes (**A1**: GR-1-BBGK-19,72 and **A2**: GR-1-BBGK-19,632) during botanical expeditions. Cutting preparation for genotype GR-1-BBGK-19,502 (**B1**) and GR-1-BBGK-19,72 (**B2**). Representative rooted cutting of GR-1-BBGK-19,72 (**C1**) and cutting that failed to root of GR-1-BBGK-19,72 (**C2**). Transplanted well-rooted plants under ex situ conservation of genotype GR-1-BBGK-19,72 (**D1**) and GR-1-BBGK-19,753 (**D2**). Bars in photos B1, B2, C1, and C2 represent 10 cm.

A variation in rooting capacity was observed between different genotypes of *C. mas*. Rooting frequencies varied from 1.19% in genotype GR-1-BBGK-19,641 with 6000 ppm IBA/softwood cuttings in summer to 69.93% in genotype GR-1-BBGK-19,638 with 10,000 ppm IBA/softwood cuttings in late summer which was the highest rooting capacity observed (*p* < 0.05). The next best rooting capacity (58.33%) was observed in genotype GR-1-BBGK-19,753 with 4000 ppm IBA/softwood cuttings in early autumn (Table 3, *p* < 0.05). Hardwood, dormant cuttings during the winter presented generally low rooting rates which took months to be reached since cuttings remained under mist throughout winter and started to root in the following spring. The highest rooting frequency observed within this group was 20.93% (*p* < 0.05) in genotype GR-1-BBGK-19,198. For the rest of the genotypes that were tested via softwood non-dormant cuttings during the summer through early autumn, rooting was still diverse, but more cases of higher rates were found compared to genotypes tested in winter, with three and two genotypes presenting >30% and 50% rooting, respectively (Table 3, *p* < 0.05, Figure 2). 

### 2.4. Multifaceted Evaluation of Greek Native Cornus mas Genotypes

An overview of the sustainable exploitation potential of the native *C. mas* genotypes assessed in this study is summarized in Table 4 in a multifaceted way based on the obtained molecular authentication results, the propagation success achieved, and the phytochemical profiles assessed. From the effectively authenticated genotypes, GR-1-BBGK-19,72 showed very high antioxidant activity and average propagation potential (Table 4, Figure 2) and could be prioritized for further research. However, genotypes GR-1-BBGK-19,190 and GR-1-BBGK-19,195 also showed high antioxidant activity but it is unclear from the current data whether the low propagation success observed was due to genotype effect or due to winter hardwood cuttings since these genotypes were only tested during the winter in contrast with GR-1-BBGK-19,72 (Table 1, Table 3, Table 5). As far as the rest of the genotypes are concerned, genotype GR-1-BBGK-19,638 stands out with very promising phytochemical potential and high propagation success and, as such, this genotype merits further investigation. Another genotype that showed a promising potential is GR-1-BBGK-19,753 with high propagation success and strong antioxidant activity (Table 3, Figure 2).

## 3. Discussion

### 3.1. Molecular Authentication of Greek Native Genotypes of Cornus mas

In general, DNA barcoding may offer support to classical morphological identification of samples and insight into phylogenetic relationships of closely related species; thus it is an efficient method for the discernment of various genotypes independently from the stage of plant development [6,10]. Herein, we provide the first-ever report regarding the molecular authentication of Greek native germplasm of *Cornus mas*, with the NJ (Neighbor-Joining) tree classification obtained from the ITS2 barcoding discerning clearly the DNA fingerprints of the Greek genotypes. Undoubtedly, more genotypes from different habitats in different regions of Greece should be evaluated to further confirm this distinctiveness. Regardless, as reported herein, the ITS2 gene can be an effective marker for the identification of *Cornus* spp. and of different genotypes thereof; thus, offering to deciphering their evolutionary relationships.

### 3.2. Nutraceutical Potential of Greek Native Genotypes of Cornus mas

The current work presents for the first time comprehensive phytochemical data for an extended range of Greek native wild-growing *Cornus mas* genotypes assessing their potential as a functional food or superfood for ex situ conservation and future breeding efforts. The parameters assessed herein revolve around human oxidative stress and particularly plant secondary metabolites with known free radical scavenging activity such as phenolic compounds and flavonoids coupled with vitamin C content [41]. The results showed that at least five Greek genotypes with significant nutraceutical potential can be distinguished, namely GR-1-BBGK-19,72, GR-1-BBGK-19,190, GR-1-BBGK-19,195, GR-1-BBGK-19,638, and GR-1-BBGK-19,753 (Table 4). Similarly to the current results, a wide range of TPC of the fruits was detected in studies dealing with wild-growing *C. mas* germplasm in Romania, coupled with significant variability among genotypes in other fruit phytochemical traits [42,43]. Some similarities can also be found between *C. mas* wild-growing genotypes of Serbia with those studied herein in terms of vitamin C content that also show higher sugar content due to higher fruit maturity index at the time of collection [15].

Although variable among genotypes, the phytochemical properties of the Greek germplasm are generally similar with both wild-growing and cultivated *C. mas* genotypes reported from countries at a similar latitudinal range as Greece such as Turkey [13]. However, studies on Central European genotypes of *C. mas* cultivated in higher latitudes have shown higher antioxidant activity (AA) of the fruits that ranged from 3.30 to 9.54 g AAE/1000 g, which implies an interaction of genotype with climatic conditions affecting fruit phytochemical profile [14]. Evidence regarding the effects of the climatic conditions in interaction with genotype on fruit phytochemical profile can also be seen through the significant correlation of total dissolved solids (TDS) with vitamin C content observed among genotypes herein, since TDS is physiologically connected to climatic factors [14]. In a more recent study concerning an Italian cultivated genotype of *C. mas*, total phenolic content (TPC) was found at 196.68 mgGAE/100 g [44], which is similar to some genotypes assessed herein and even lower than three of the studied genotypes, while vitamin C content was at similar ranges. Recently, the phytochemical profile of fruits was comparatively evaluated between cultivars of *C. mas*, genotypes of the Chinese endemic *Cornus officinalis* Siebold and Zucc., and hybrids thereof (*C. mas* X *C. officinalis*). *C. mas* cultivars in that study have shown a similar TPC range with the studied Greek genotypes apart from one cultivar which was higher, yet the TPC and AA of the hybrids was generally higher, indicating the significance of conservation of *C. mas* germplasm stocks for breeding programs [22]. The nutraceutical potential of native genotypes has been similarly assessed on other Greek germplasm resources such as, for example, *Rosa canina* [10] with slightly higher potential of AA and TPC detected and comparable to some extent with that of *C. mas*, but with very high content of vitamin C for *R. canina* [10]. 

The climatic conditions (most prominently temperature along with sunshine) during the last stages of fleshy fruit ripening in cornelian cherries may affect acid reduction and sugar increase, which in turn affects biosynthesis of secondary metabolites [45,46]. As such, the climatic conditions of the natural habitats of wild populations on one hand and the stage of fruit collection and fruits’ ripening status on the other hand are considered as significant factors in terms of comparative phytochemical evaluations. However, in this study no significant correlations were observed between the fruit phytochemical properties measured and the altitude from which they were collected in the wild or their maturity index determined as the ratio of sugar content to malic acid content. This observation indicates that the variability of phytochemical properties observed among Greek native genotypes of *C. mas* may be attributed to genetic factors rather than timing of fruit harvest or altitudinal differences between collection sites. Nevertheless, further ecological profiling work in terms of correlation studies with more environmental/topoclimatic parameters and more fruit phenological traits on Greek *C. mas* wild populations is suggested. 

### 3.3. Propagation Potential of Greek Native Genotypes of Cornus mas

A further component of the multifaceted evaluation of wild Greek *C. mas* genotypes investigated herein is the asexual propagation via cuttings which was conducted for the first time in Greek germplasm. Similarly to fruit phytochemical data, the genotypes of *C. mas* tested showed variable rooting capacity with a number of them being below the commercially accepted threshold [47]. In all cases, however, external hormone application was necessary to achieve even low rooting frequencies which are in agreement with similar studies [38,39,48]. Hardwood dormant cuttings tested in the current study remained under mist for the duration of winter and started to root the following spring giving low rooting rates (<25%) under 10,000 ppm IBA treatment. This observation concurs with similar studies on cutting propagation of *C. mas* genotypes in other countries where softwood cuttings surpassed hardwood cuttings under the 10,000 ppm IBA treatment [38]. The poor results on hardwood cuttings indicate that it is not economically efficient to produce new plants via winter dormant cuttings [37,40], a fact that also applies for the Greek germplasm. Softwood cutting material with a small degree of lignification has known advantages in rooting quality with external hormone utilization [49], producing higher rooting rates in *Cornus alba* [50]. Even in other genera, cutting type affected by the status of mother’s plant growth has been linked to rooting capacity and quality [51,52,53]. Consequently, since only two of the selected genotypes evaluated herein showed appreciable propagation potential (>50% rooting in genotypes GR-1-BBGK-19,638 and GR-1-BBGK-19,753) with softwood cuttings (Table 3), further research is required to explore pathways on improving rooting capacity but also to investigate the relationships between cutting type and rooting capacity and quality in more detail. Additionally, further research is proposed on the correlation of rooting factors with early plant establishment and survival in *C. mas* propagation since such a correlation has been shown to be significant in other Greek native germplasm resources such as *Sambucus nigra* [6]. 

Finally, asexual propagation through grafting has been attempted on European *C. mas* germplasm with positive results [54], showing that successful grafting in *C. mas* can enhance growth and development of the scion [55]. For the Greek native *C. mas* genotypes studied herein, the method of grafting a genotype with high phytochemical potential but low rooting capacity onto another genotype with improved rooting capacity may provide a good option in dealing with difficult-to-root genotypes but with valuable fruit traits. However, further research is required to systematically test this strategy. 

## 4. Materials and Methods

### 4.1. Collection and Documentation of Plant Material

**Figure 3:**
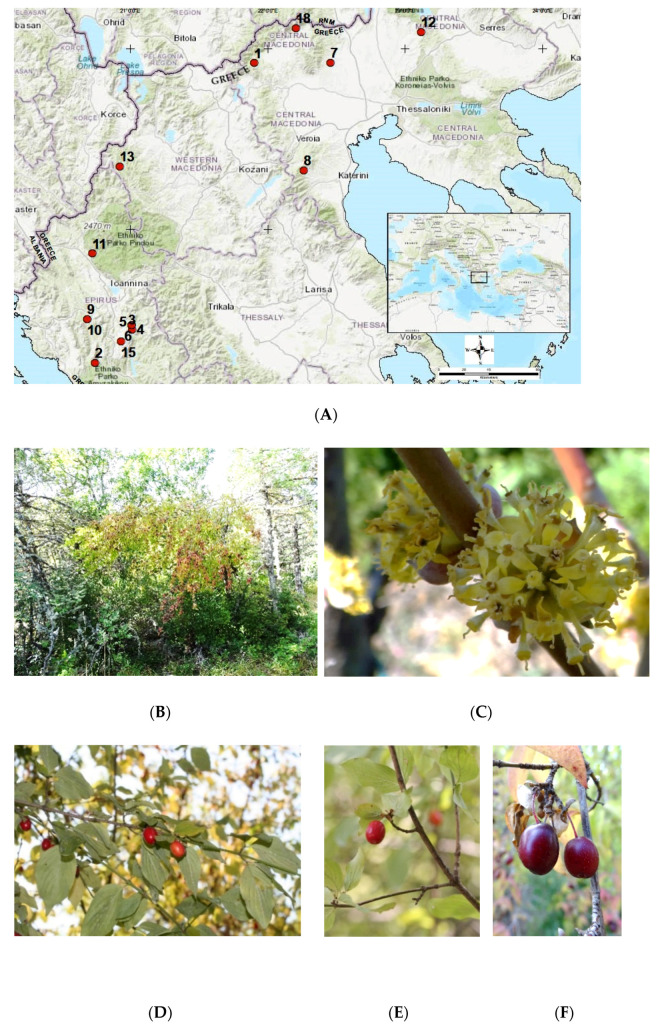
(**A**) Geographical distribution of the *Cornus mas* Greek native genotypes sampled for taxonomic identification, DNA barcoding, phytochemical assessment, and asexual propagation trials. (**B**) Typical habit of wild-growing individuals; morphology of flowers (**C**) leaves and fruits (**D**) in early (**E**) and full ripening (**F**).

A plethora of botanical expeditions took place covering a wide range of geographically distant and altitudinally different natural habitats of *C. mas* across Northern Greece reached at consecutive stages across two years (Table 5). Targeted and variable types of plant material were collected (Table 5): (i) leaves from 20 individuals from each genotype for DNA analysis; (ii) hardwood dormant twigs for propagation from six genotypes (winter 2018); (iii) softwood stem cuttings for propagation from 14 genotypes (14 in spring–late summer and 5 in autumn of 2019); (iv) ripe fruits from nine genotypes for phytochemical evaluation (autumn 2019). The work resulted in the documentation of 18 *C. mas* genotypes (population samples) in total from healthy wild-growing individuals (Figure 3). After each expedition, the collected material was promptly transferred to the laboratory where it was taxonomically identified [56] and was assigned with a unique IPEN (International Plant Exchange Network) accession number given by the Balkan Botanic Garden of Kroussia of the Institute of Plant Breeding and Genetic Resources (IPB&GR) of the Hellenic Agricultural Organization-Demeter (ELGO-Demeter). Plant material collection in all cases was conducted under a special permit to the IPB&GR, ELGO-Demeter (Permit 82336/879 of 18/5/2019 and 26895/1527 of 21/4/2021) issued by the Greek Ministry of Environment and Energy. The overall work was conducted under the auspices of the research project “Highlighting local traditional varieties and wild native forest fruit trees and shrubs” (acronym: EcoVariety, Τ1ΕΔΚ-05434). 

### 4.2. DNA Isolation 

A standardized commercial DNA extraction kit (Nucleospin Plant II, Macherey-Nagel) was used for DNA extraction following the manufacturer’s instructions using approximately 30 mg of dried leaf sample from each *C. mas* genotype that was previously ground in liquid nitrogen. 

### 4.3. Polymerase Chain Reaction (Pcr) Amplification and Sequence Analysis

PCR amplification was conducted according to [57] using one primer set of the nuclear ITS2 barcode region [58]. The resulting PCR products for each genotype were consecutively sequenced using an automated ABI 3730 sequencer (PE Applied Biosystems), in two directions of each fragment with a Big Dye terminator v3.1 Cycle sequencing kit (PE Applied Biosystems, Foster City, CA, USA). The sequences were aligned using the CLUSTAL W program. 

### 4.4. Molecular Data Analysis

Following the results of the sequence analysis, the molecular authentication of the *Cornus mas* genotypes studied was conducted using three methods: (i) evaluation against the nucleotide database at NCBI using the Basic Local Alignment Search Tool (BLAST); (ii) using maximum-likelihood models for the genetic divergence method; and (iii) tree topology analysis using the Neighbor-Joining (NJ) method based on different loci in MEGAX with the K2P distance model and 1000 bootstrap replications. The generated ITS2 DNA barcoding sequences (without primers used for PCR amplification) were deposited to the NCBI-Genbank (https://www.ncbi.nlm.nih.gov/BankIt/, accessed 5 May 2022) under the accession numbers MZ35480-MZ35488. 

### 4.5. Phylogenetic Relationships 

The phylogenetic relationship of *Cornus* spp. was inferred using the Neighbor-Joining (NJ) method [59]. The percentage of replicate trees in which the associated taxa clustered together in the bootstrap test (1000 replicates) are shown next to the branches [60]. The evolutionary distances were estimated using the Maximum Composite Likelihood (MCL) method [61]. All ambiguous positions were removed for each sequence pair (pairwise deletion option). The phylogenetic dendrogram was constructed using the MEGA X software [62].

### 4.6. Propagation Trials 

Depending on the propagation material obtained from each expedition, a variety of targeted experiments were conducted using different external hormone application treatments of indole-3-butyric acid (IBA) [50,63] and different cutting types taken at different seasons from varying stages of mother plant growth (Table 5). Cuttings were set for rooting in propagation trays under mist where relative humidity (RH) was maintained at >85% within a greenhouse at ambient temperature. The substrate used was peat (Klasmann, KTS 1):perlite at 1:3 *v/v*. Cuttings were attended weekly to assess their rooting capacity. The produced mother plants were kept ex situ at the laboratory’s nursery in the grounds of IPB and GR under ambient conditions for plant adaptation. The plants were watered regularly and were grown in 3 L pots using a mixture of peat (Klasmann, KTS 2) and perlite (3:1, *v/v*).

### 4.7. Phytochemical Analysis of Cornus mas Fruits 

A modified method described in [64] was used for preparation of the extracts. An appropriate volume of MeOH/H_2_O (60:40) was mixed with 2–5 g of homogenized sample and was centrifuged at 4000rpm at 4°C. The collected supernatant was made up to 20 mL volume and was used for the determination of total phenolic content (TPC), total flavonoids (TF), and antioxidant activity (AA). For the determination of TPC, phenolic extract 0.20 mL along with 2.3 mL of H_2_O and 0.25 mL of Folin–Ciocalteu reagent were added in a volumetric flask [64] in which, after 3 min, 0.50 mL of 20% Na_2_CO_3_ was added, and the volume was made up to 5 mL. The above solution was stored in a dark place for 2 h and the absorbance was measured at 725 nm against blank solution. The calculation of TPC was conducted using a standard curve of gallic acid at various concentrations giving the results expressed as gallic acid equivalents (GAE)/100 g of sample. All analyses were carried out in triplicate. 

A modified method for the determination of TF was used according to [65]. In a test tube, 5 μL of the above extract were added along with 3270 μL of H_2_O and 75 μL of 5% NaNO_2_, stirred, and stored in the dark for 5 min which was then added with 150 μL of 10% AlCl_3_-6H_2_O, mixed, and stored in the dark for a further 6 min. Consequently, it was added with 500 μL of 1 M NaOH and the absorbance was measured at 510 nm against H_2_O as blank. The calculation of TF was conducted using a standard curve of catechin at various concentrations giving results expressed as catechin equivalents (CE)/100 g of sample. All analyses were carried out in triplicate.

A modified method for the determination of AA was employed described in [66]. In a 5 mL plastic cuvette, 0.1 mL of phenolic extract was added along with 2.9 mL 0.10 mM (2,2-diphenyl-1-picrylhydrazyl (DPPH)) in MeOH, stored in the dark for 15 min and then the absorbance was measured at 517 nm against MeOH as blank. The control sample was prepared using 0.10 mM DPPH in MeOH. The percentage of radical scavenging activity (%RSA) was carried out in triplicate and was calculated using the following equation:

$$\%\mathrm{RSA}=\frac{A_{o}-A_{s}}{A_{o}}*100$$

where 

*A_o_* = absorbance of control sample.

*A_s_* = absorbance of the sample after 15 min of incubation.

A modified method for the determination of vitamin C presented in [67] was used. A mixture of homogenized sample (2–5 g) and 5 mL of 4.5% *w/v* metaphosphoric acid (MPA) solution was stirred and centrifuged at 8000 rpm at 4°C for 20 min. The supernatant (1 mL) was taken and diluted up to 10 mL with 4.5% MPA solution and it was filtered through 0.45 μm polyethersulfone filters. The vial was covered with aluminum foil to prevent oxidation of ascorbic acid and stored at 4 °C until HPLC-DAD analysis. HPLC-DAD analysis conditions: Column (Agilent Eclipse XDB-C18) 4.6 mm × 150 mm, 5μm, elution solvent: aqueous 0.005 M H_2_SO_4_ solution at a flow rate of 0.5 mL/min (isocratic) and wavelength 245 nm. A standard curve of ascorbic acid at various concentrations was used for vitamin C content calculation which gave results expressed as ascorbic acid equivalents (AAE)/100 g of sample. 

Sugar content of fruit samples was measured via Brix analysis (^o^Bx), and the maturity index (MI) of fruits was calculated as the ratio of sugar content in °Bx to malic acid (MA) content expressed as grams of MA per 100 g.

All analyses were carried out in triplicate. 

### 4.8. Experimental Design and Statistical Analysis of Propagation and Phytochemical Data 

The propagation trials followed a complete randomized design. The number of replicate cuttings per treatment varied according to the volume of material obtained from each expedition (Table 5). Targeted IBA hormone application treatments were applied ranging from 2000 to 10,000 ppm depending on the season and whether the cuttings were softwood or hardwood, respectively (Table 5). Each trial included a control with an equal number of replicate cuttings. The rooting frequencies obtained were compared through pairwise Pearson Chi-Square tests. 

For the phytochemical data that were measured in triplicate, a mean coupled with its standard deviation (±S.D.) was calculated in each case and means were compared using Tukey’s HSD post hoc test. In addition, the phytochemical data were subjected to Pearson’s correlation coefficient analysis to determine the correlations between the different fruit phytochemical traits measured along with the differences in altitude between the natural habitats of *C. mas* that collection occurred in. All analyses were conducted using the IBM-SPSS 23.0 software. 

## 5. Conclusions

In conclusion, the multifaceted evaluation that was conducted provides a first depiction of the sustainable exploitation potential of Greek native *Cornus mas* genotypes. All genotypes collected from the wild are under ex situ conservation at the IPB and GR for future monitoring, since juvenile–mature correlations are known in perennial crops from the literature [68,69]; such aspects should be taken into account for future long-term breeding programs. The transition from the juvenile to the mature (producing) stage followed by several phenological and developmental changes was found to vary among genotypes mainly in forest species but also in cultivated deciduous tree species with stone fruits such as apples [69]. Consequently, further work on molecular authentication, ecological profiling, and phytochemical profiling is proposed coupled with further research on propagation. In addition, the long-term monitoring of the current Greek genotypes is needed, and in fact, pilot cultivation trials of the genotypes prioritized herein have been established (ongoing process). The data obtained in the current study along with the conservation of the plant material collected can serve as a basis for future breeding efforts, creating for the first time a framework for the sustainable utilization of Greek native *C. mas* germplasm as a superfood with significant agronomic potential.

## Figures and Tables

**Table 1:** Total phenolic content (TPC), antioxidant activity (AA), total flavonoids (TF), and vitamin C content assessed in fruit samples of wild-growing Greek genotypes of *Cornus mas*.

Population Sample	AA (%RSA)	TPC (mgGAE/100 g)	Vitamin C (mgAAE/100 g)	TF (mgCE/100 g)
GR-1-BBGK-19,72	90.99 ± 0.15 ^g^	54.21 ± 0.1 ^e^	1.03 ± 0.01 ^a^	0.19 ± 0.01 ^ab^
GR-1-BBGK-19,190A	80.48 ± 3.08 ^d^	304.73 ± 6.56 ^i^	21.22 ± 0.05 ^c^	0.73 ± 0.12 ^de^
GR-1-BBGK-19,190B	84.07 ± 0.01 ^e^	49.29 ± 0.01 ^cd^	33.48 ± 0.01 ^f^	0.22 ± 0.1 ^ab^
GR-1-BBGK-19,195	86.56 ± 0.01 ^ef^	49.43 ± 0.01 ^cde^	32.73 ± 0.01 ^f^	0.15 ± 0.01 ^a^
GR-1-BBGK-19,590	94.72 ± 0.15 ^hi^	82.74 ± 0.31 ^g^	1.31 ± 0.01 ^a^	0.44 ± 0.01 ^bc^
GR-1-BBGK-19,632	85.84 ± 0.01 ^ef^	47.58 ± 0.01 ^c^	28.13 ± 0.01 ^e^	0.19 ± 0.01 ^ab^
GR-1-BBGK-19,633A	95.52 ± 0.01 ^hi^	52.31 ± 0.01 ^cde^	44.69 ± 0.06 ^j^	0.49 ± 0.01 ^cd^
GR-1-BBGK-19,633B	90.5 ± 1.28 ^g^	195.2 ± 0.0 ^h^	37.18 ± 0.91 ^g^	0.28 ± 0.06 ^abc^
GR-1-BBGK-19,638A	55.46 ± 0.01 ^a^	29.93 ± 0.01 ^a^	52.33 ± 0.71 ^k^	0.44 ± 0.01 ^bc^
GR-1-BBGK-19,638B	94.01 ± 0.48 ^hi^	337.14 ± 0.0 ^j^	58.97 ± 0.9 ^l^	0.44 ± 0.09 ^bc^
GR-1-BBGK-19,641	95.64 ± 0.1 ^i^	80.43 ± 0.14 ^g^	41.33 ± 0.03 ^i^	0.49 ± 0.03 ^cd^
GR-1-BBGK-19,669	87.52 ± 0.1 ^f^	355.46 ± 0.01 ^k^	23.27 ± 0.26 ^d^	0.86 ± 0.09 ^e^
GR-1-BBGK-19,753	70.11 ± 0.05 ^c^	40.56 ± 0.15 ^b^	1.26 ± 0.02 ^a^	0.19 ± 0.02 ^ab^
GR-1-BBGK-19,844	95.94 ± 0.01 ^i^	52.63 ± 0.01 ^de^	40.05 ± 1.51 ^hi^	0.17 ± 0.1 ^a^
GR-1-BBGK-19,847	65.07 ± 0.01 ^d^	40.69 ± 0.05 ^b^	0.95 ± 0.1 ^a^	0.11 ± 0.1 ^a^
GR-1-BBGK-19,848	57.9 ± 0.02 ^a^	38.82 ± 0.05 ^b^	39.13 ± 0.1 ^h^	0.17 ± 0.1 ^a^
GR-1-BBGK-19,926	94.43 ± 0.02 ^hi^	73.61 ± 0.02 ^f^	15.96 ± 0.2 ^b^	0.21 ± 0.2 ^ab^

Values represent mean values ± standard deviation (S.D.) of samples analyzed in triplicate (*n* = 3); values within the same column that do not share the same letter are significantly different (Tukey post-hoc test, *p* < 0.05). For genotypes GR-1-BBGK-19,190, GR-1-BBGK-19,633, and GR-1-BBGK-19,638, capital letters A and B denote two consecutive years that fruits were measured.

**Table 2:** Pearson’s correlation coefficients for the Greek *C. mas* genotypes (*n* = 14) between altitudes (m), four fruit nutraceutical properties assessed namely total phenolic content (TPC, mgGAE/100 g), antioxidant activity (AA, %RSA), total flavonoids (TF, mgCE/100 g), and vitamin C content (Vit C, mgAAE/100 g) as well as two complementary fruit phytochemical properties measured, i.e., maturity index (MI) expressed as the ratio of sugar content (°Brix) to malic acid content (gMA/100 g) and total dissolved solids (TDS, mg/L).

	Altitude	TDS	MI	AA	TPC	Vit C	TF
Altitude		−0.052 (0.861)	−0.375 (0.187)	−0.019 (0.949)	0.241 (0.406)	0.080 (0.787)	0.223 (0.444)
TDS			0.475 (0.086)	0.095 (0.746)	0.132 (0.654)	0.600 * (0.023)	0.172 (0.556)
MI				0.238 (0.413)	−0.184 (0.529)	0.326 (0.255)	0.060 (0.838)
AA					0.091 (0.758)	−0.295 (0.307)	0.021 (0.944)
TPC						−0.082 (0.781)	0.843 ** (0.000)
Vit C							0.188 (0.521)
TF							

Respective *p*-values are shown in parentheses. ** Correlation is significant at the 0.01 level (2-tailed). * Correlation is significant at the 0.05 level (2-tailed).

**Table 3:** Overview of the propagation results achieved in terms of highest rooting frequencies after experimental setups in different seasons (winter 2018, spring–late summer 2019, and early–late autumn 2019) with different types of initial material (softwood or hardwood cuttings) collected directly from wild-growing Greek native populations of *Cornus mas* (18 genotypes).

Genotype (Accession Number)	Season	Cutting Type	Hormone Treatment (ppm IBA)	Rooting (%)
GR-1-BBGK-19,72	Summer	Softwood	4000	33.30 ^‡^
GR-1-BBGK-19,190	Winter	Hardwood	10,000	13.29 ^†^
GR-1-BBGK-19,195	Winter	Hardwood	10,000	1.39
GR-1-BBGK-19,196	Winter	Hardwood	10,000	8.33
GR-1-BBGK-19,197	Winter	Hardwood	10,000	2.86
GR-1-BBGK-19,198	Winter	Hardwood	10,000	20.93 ^†^
GR-1-BBGK-19,502	Summer	Softwood	4000	18.60
GR-1-BBGK-19,590	Early autumn	Softwood	4000	16.67
GR-1-BBGK-19,632	Summer	Softwood	5000	20.41
GR-1-BBGK-19,633	Summer	Softwood	5000	2.27
GR-1-BBGK-19,638	Late summer	Softwood	10,000	69.33 ^‡^
GR-1-BBGK-19,641	Late summer	Softwood	6000	1.19
GR-1-BBGK-19,669	Summer	Softwood	2000	28.60
GR-1-BBGK-19,753	Late summer	Softwood	4000	58.33 ^‡^
GR-1-BBGK-19,844	Early autumn	Softwood	4000	33.33 ^‡^
GR-1-BBGK-19,847	Early autumn	Softwood	4000	31.94
GR-1-BBGK-19,848	Autumn	Hardwood	6000	8.33
GR-1-BBGK-19,926	Autumn	Hardwood	4000	10.34

The symbols † and ‡ denote the highest rooting frequencies for hardwood and softwood cuttings, respectively, following pairwise comparisons of the observed rooting frequencies via Pearson X^2^ tests. All cases were tested against a control treatment with no hormone application and no rooting.

**Table 4:** Multifaceted evaluation of Greek native *Cornus mas* genotypes based on molecular authentication effectiveness (Figure 1), fruit phytochemical potential expressed as antioxidant activity (AA, %RSA: low ≤50, average 51–70, high 71–90, very high >90), total phenolic content (TPC, mg GAE/100 g: low ≤50, average 51–100, high 101–200, very high >200) and vitamin C content (mg AAE/100 g: low ≤30, average 31–50, high >50) (Table 1), and asexual propagation potential expressed as hormone-induced rooting under the most successful application (very low < 10%, low 11–30%, average 31–55%, or high >55%), (Table 3).

IPEN Accession Number	DNA Barcoding	Phytochemical Potential			Propagation Potential
		AA (%RSA)	TPC (mg GAE/100 g)	Vitamin C (mg AAE/100 g)	
GR-1-BBGK-19,72	Effective	Very high	Average	Low	Average
GR-1-BBGK-19,190	Effective	High	Very high	Average	Low
GR-1-BBGK-19,195	Effective	High	Low	Average	Very low
GR-1-BBGK-19,196	Effective	-	-	-	Very low
GR-1-BBGK-19,197	Effective	-	-	-	Very low
GR-1-BBGK-19,198	Effective	-	-	-	Low
GR-1-BBGK-19,502	Effective	-	-	-	Low
GR-1-BBGK-19,590	Easy	Very high	Average	Low	Low
GR-1-BBGK-19,632	Easy	High	Low	Low	Low
GR-1-BBGK-19,633	Easy	Very high	High	Average	Very low
GR-1-BBGK-19,638	Easy	Very high	Very high	High	High
GR-1-BBGK-19,641	Easy	Very high	Average	Average	Very low
GR-1-BBGK-19,669	Easy	High	Very high	Low	Low
GR-1-BBGK-19,753	Easy	High	Low	Low	High
GR-1-BBGK-19,844	Easy	Very high	Average	Average	Average
GR-1-BBGK-19,847	Easy	Average	Low	Low	Average
GR-1-BBGK-19,848	Easy	Average	Low	Average	Very low
GR-1-BBGK-19,926	Easy	Very high	Average	Low	Low

**Table 5:** IPEN accession number, location, altitude, and sampling details of the genotypes (population samples) of Greek native *Cornus mas* germplasm collected from various areas and habitats of Northern Greece in 2018–2019.

No	IPEN Accession Number	Greek Prefecture	Area	Coordinates (HGRS87/EGSA87) (Lat, Lon)	Altitude (m)	1st Sampling (Winter 2018)	2nd Sampling (Spring–Late Summer 2019)	3rd Sampling (Autumn 2019)
1	GR-1-BBGK-19,72	Central Macedonia	Pella, Aridea	40.919338, 21.900725	882	HWSC	SWSC, LS	RFS
2	GR-1-BBGK-19,190	Epirus	Preveza, Kranea	39.248017, 20.742179	513	HWSC	LS	RFS
3	GR-1-BBGK-19,195	Epirus	Ioannina, Dafni	39.43568, 21.015093	447	HWSC	LS	RFS
4	GR-1-BBGK-19,196	Epirus	Ioannina, Xirovouni	39.461535, 21.008474	1070	HWSC	SWSC, LS	
5	GR-1-BBGK-19,197	Epirus	Ioannina, Xirovouni	39.461535, 21.008474	1070	HWSC	LS	
6	GR-1-BBGK-19,198	Epirus	Ioannina, Xirovouni	39.461535, 21.008474	1070	HWSC	LS	
7	GR-1-BBGK-19,502	Central Macedonia	Kilkis, Goumenissa	40.92079, 22.45934	170		SWSC, LS	
8	GR-1-BBGK-19,590	Central Macedonia	Pieria, Elatochori	40.32734, 22.26310	780		SWSC	SWSC, RFS
9	GR-1-BBGK-19,632	Epirus	Ioannina, Dodoni	39.49421, 20.68520	500		SWSC, RFS	
10	GR-1-BBGK-19,633	Epirus	Ioannina, Dodoni	39.49421, 20.68520	550		SWSC, RFS	
11	GR-1-BBGK-19,638	Epirus	Ioannina, Zagori	39.86281, 20.72305	960		SWSC, RFS	
12	GR-1-BBGK-19,641	Central Macedonia	Kilkis, Pontokerasia	41.08850, 23.117975	648		SWSC, RFS	
13	GR-1-BBGK-19,669	West Macedonia	Kastoria, Mt Grammos	40.34888, 20.92475	1165		SWSC, RFS	
14	GR-1-BBGK-19,753	East Macedonia	Drama, Ahladomelea	41.41379, 24.00165	590			SWSC, RFS
15	GR-1-BBGK-19,844	Epirus	Preveza, Anaogeio	39.37108, 20.93397	1100			SWSC, RFS
16	GR-1-BBGK-19,847	Thrace	Xanthi, Ano kalyva	41.29185, 24.62509	620			SWSC, RFS
17	GR-1-BBGK-19,848	East Macedonia	Drama, Silli	41.35046, 24.52971	750			SWSC, RFS
18	GR-1-BBGK-19,926	Central Macedonia	Pella, Notia	41.11128, 22.20423	880			SWSC, RFS

RFS: Ripe cornelian cherry fruits sample for chemical analysis; HWSC: hardwood stem cuttings; SWSC: softwood stem cuttings for propagation; LS: leaf samples for DNA analysis

## Data Availability

All data supporting the results of this study are included in the manuscript and datasets are available upon request.

## Abbreviations

All abbreviations are explained at their first mention in the manuscript.

## References

[1] Krigas N., Tsoktouridis G., Anestis I., Khabbach A., Libiad M., Megdiche-Ksouri W., Ghrabi-Gammar Z., Lamchouri F., Tsiripidis I., Tsiafouli M.A. (2021). Exploring the potential of neglected local endemic plants of three Mediterranean regions in the ornamental sector: Value chain feasibility and readiness timescale for their sustainable exploitation. Sustainability.

[2] Ercisli S. (2004). A short review of the fruit germplasm resources of Turkey. Genet. Resour..

[3] Verma N., Mohanty A., Lal A. (2010). Pomegranate genetic resources and germplasm conservation: A Review. Fruit Veg. Cereal Sci. Biotechnol..

[4] Botu M., Botu I., Achim G., Preda S., Scutelnicu A., Giura S. (2017). Conservation of fruit tree genetic resources and their use in the breeding process. Ann. Valahia Univ. Targoviste.

[5] Manco R., Basile B., Capuozzo C., Scognamiglio P., Forlani M., Rao R., Corrado G. (2019). Molecular and phenotypic diversity of traditional European plum (*Prunus domestica* L.) germplasm of Southern Italy. Sustainability.

[6] Karapatzak E., Dichala O., Ganopoulos I., Karydas A., Papanastasi K., Kyrkas D., Yfanti P., Nikisianis N., Fotakis D., Patakioutas G. (2022). Molecular authentication, propagation trials and field establishment of Greek native genotypes of *Sambucus nigra* L. (Caprifoliaceae): Setting the basis for domestication and sustainable utilization. Agron. J..

[7] Cosmulescu S., Trandafir I., Nour V. (2017). Phenolic acids and flavonoids profiles of extracts from edible wild fruits and their antioxidant properties. Int. J. Food Prop..

[8] Che C.T., Zhang H. (2019). Plant natural products for human health. Int. J. Mol..

[9] Durazzo A., Lucarini M., Souto E.B., Cicala C., Caiazzo E., Izzo A.A., Novellino E., Santini A. (2019). Polyphenols: A concise overview on the chemistry, occurrence, and human health. Phytother. Res..

[10] Maloupa E., Karapatzak E., Ganopoulos I., Karydas A., Papanastasi K., Kyrkas D., Yfanti P., Nikisianis N., Zahariadis A., Kosma I.S. (2021). Molecular authentication, phytochemical evaluation and asexual propagation of wild-growing *Rosa canina* L. (Rosaceae) genotypes of Northern Greece for sustainable exploitation. Plants.

[11] Da Ronch F., Caudullo G., Houston Durrant T., de Rigo D., San-Miguel-Ayanz J., de Rigo D., Caudullo G., Houston Durrant T., Mauri A. (2016). *Cornus mas* in Europe: Distribution, habitat, usage and threats. The European Atlas of Forest Tree Species.

[12] Baumann H. (1993). The Greek Plant World in Myth, Art and Literature.

[13] Tural S., Koca I. (2008). Physico-chemical and antioxidant properties of Cornelian cherry fruits (*Cornus mas* L.) grown in Turkey. Sci. Hortic..

[14] Rop O., Mlcek J., Kramarova D., Jurikova T. (2010). Selected cultivars of Cornelian cherry (*Cornus mas* L.) as a new food source for human nutrition. Afr. J. Biotechnol..

[15] Bijelić S.M., Gološin B.R., Ninić Todorović J.I., Cerović S.B., Popović B.M. (2011). Physicochemical fruit characteristics of Cornelian cherry (*Cornus mas* L.) genotypes from Serbia. HortScience.

[16] Szczepaniak O.Μ., Kobus-Cisowska J., Kusek W., Przeor M. (2019). Functional properties of Cornelian cherry (*Cornus mas* L.): A comprehensive review. Eur. Food Res. Technol..

[17] Tiptiri-Kourpeti A., Fitsiou E., Spyridopoulou K., Vasileiadis S., Iliopoulos C., Galanis A., Vekiari S., Pappa A., Chlichlia K. (2019). Evaluation of antioxidant and antiproliferative properties of *Cornus mas* L. fruit juice. Antioxidants.

[18] Moldovan B., Filip A., Clichici S., Suharoschi R., Bolfa P., David L. (2016). Antioxidant activity of Cornelian cherry (*Cornus mas* L.) fruits extract and the in vivo evaluation of its anti-inflammatory effects. J. Funct. Foods.

[19] Bayram H.M., Ozturkcan S.A. (2020). Bioactive components and biological properties of Cornelian cherry (*Cornus mas* L.): A comprehensive review. J. Funct. Foods.

[20] Radbeh Z., Asefi N., Hamishehkar H., Roufegarinejad L., Pezeshki A. (2020). Novel carriers ensuring enhanced anti-cancer activity of *Cornus mas* (Cornelian cherry) bioactive compounds. Biomed. Pharmacother..

[21] Przybylska D., Kucharska A.Z., Cybulska I., Sozański T., Piórecki N., Fecka I. (2020). *Cornus mas* L. stones: A valuable by-product as an ellagitannin source with high antioxidant potential. Molecules.

[22] Klymenko S., Kucharska A.Z., Sokół-Łętowska A., Piórecki N., Przybylska D., Grygorieva O. (2021). Iridoids, flavonoids, and antioxidant capacity of *Cornus mas*, *C. officinalis*, and *C. mas* × *C. officinalis* fruits. Biomolecules.

[23] Brindza P., Brindza J., Tóth D., Klimenko S.V., Grigorieva O. (2007). Slovakian Cornelian cherry (*Cornus mas* L.): Potential for cultivation. Acta Hortic..

[24] Bijelić S.M., Gološin B., Todorović J.N., Cerović S. (2011). Morphological characteristics of best Cornelian cherry (*Cornus mas* L.) genotypes selected in Serbia. Genet. Resour. Crop Evol..

[25] Yilmaz K.U., Ercisli S., Zengin Y., Sengul M., Kafkas E. (2009). Preliminary characterisation of cornelian cherry (*Cornus mas* L.) genotypes for their physico-chemical properties. Food Chem..

[26] Hamid H., Hamidoghli Y., Hajilo J., Adlipour M. (2011). Antioxidant capacity and phytochemical properties of cornelian cherry (*Cornus mas* L.) genotypes in Iran. Sci. Hortic..

[27] Moradi Y., Khadivi A., Salehi-Arjmand H. (2019). Morphological and pomological characterizations of Cornelian cherry (*Cornus mas* L.) to select the superior accessions. Sci. Hortic..

[28] Jaćimović V., Božović D., Ercisli S., Bosančić B., Necas T. (2020). Sustainable Cornelian cherry production in Montenegro: Importance of local genetic resources. Sustainability.

[29] Martinović A., Cavoski I. (2020). The exploitation of cornelian cherry (*Cornus mas* L.) cultivars and genotypes from Montenegro as a source of natural bioactive compounds. Food Chem..

[30] Hebert P.D., Cywinska A., Ball S.L., de Waard J.R. (2003). Biological identifications through DNA barcodes. Proc. Royal Soc. B.

[31] Tsoktouridis G., Krigas N., Sarropoulou V., Kampouropoulou S., Papanastasi K., Grigoriadou K., Menexes G., Maloupa E. (2019). Micropropagation and molecular characterization of Thymus sibthorpii Benth. (Lamiaceae), an aromatic-medicinal thyme with ornamental value and conservation concern. In Vitro Cell. Dev. Biol.-Plant.

[32] Yu J., Wu X., Liu C., Newmaster S., Ragupathy S., Kress W.J. (2021). Progress in the use of DNA barcodes in the identification and classification of medicinal plants. Ecotoxicol. Environ. Saf..

[33] Pipinis E., Hatzilazarou S., Kostas S., Bourgou S., Megdiche-Ksouri W., Ghrabi-Gammar Z., Libiad M., Khabbach A., El Haissoufi M., Lamchouri F. (2022). Facilitating conservation and bridging gaps for the sustainable exploitation of the Tunisian local endemic plant *Marrubium aschersonii* (Lamiaceae). Sustainability.

[34] Hatzilazarou S., El Haissoufi M., Pipinis E., Kostas S., Libiad M., Khabbach A., Lamchouri F., Bourgou S., Megdiche-Ksouri W., Ghrabi-Gammar Z. (2021). GIS-facilitated seed germination and multifaceted evaluation of the Endangered *Abies marocana* Trab. (Pinaceae) Enabling conservation and sustainable exploitation. Plants.

[35] Kostas S., Hatzilazarou S., Pipinis E., Bourgou S., Ben Haj Jilani I., Ben Othman W., Megdiche-Ksouri W., Ghrabi-Gammar Z., Libiad M., Khabbach A. (2022). DNA barcoding, GIS-facilitated seed germination and pilot cultivation of *Teucrium luteum* subsp. *gabesianum* (Lamiaceae), a Tunisian local endemic with potential medicinal and ornamental value. Biology.

[36] Gismondi A., Rolfo M.F., Leonardi D., Rickards O., Canini A. (2012). Identification of ancient *Olea europaea* L. and *Cornus mas* L. seeds by DNA barcoding. Comptes Rendus Biol..

[37] Pirlak L. (2000). Effects of different cutting times and IBA doses on the rooting rate of hardwood cuttings of cornelian cherry (*Cornus mas* L.). Anadolu J. Aegean Agric. Res. Inst..

[38] Marković M., Grbić M., Djukić M. (2014). Effects of cutting type and a method of IBA application on rooting of softwood cuttings from elite tree of cornelian cherry (*Cornus mas* L.) from Belgrade area. Silva Balc..

[39] Kosina J., Baudyšová M. (2011). Propagation of less known fruit crops by cuttings. Ved. Pr. Ovocn..

[40] Hassanpour H., Ali Shiri M. (2014). Propagation of Iranian Cornelian cherry (*Cornus mas* L.) by rooted stem cuttings. Not. Sci. Biol..

[41] Cory H., Passarelli S., Szeto J., Tamez M., Mattei J. (2018). The Role of polyphenols in human health and food systems: A mini-review. Front. Nutr..

[42] Cosmulescu S., Trandafir I., Cornescu F. (2018). Antioxidant capacity, total phenols, total flavonoids and color component of Cornelian cherry (*Cornus mas* L.) wild genotypes. Not. Bot. Horti Agrobot. Cluj-Napoca.

[43] Cosmulescu S., Cornescu F. (2020). Variability in physical and chemical characteristics of Cornelian cherry fruits (*Cornus mas* L.) from Romanian Oltenia region’s spontaneous flora and role of the climatic conditions. Braz. J. Bot..

[44] De Biaggi M., Donno D., Mellano M.G., Riondato I., Rakotoniaina E.N., Beccaro G.L. (2018). *Cornus mas* (L.) fruit as a potential source of natural health-promoting compounds: Physico-chemical characterisation of bioactive components. Plant Foods Hum. Nutr..

[45] Prasanna V., Prabha T.N., Tharanathan R.N. (2007). Fruit ripening phenomena–An overview. Crit. Rev. Food Sci. Nutr..

[46] Moretti C.L., Mattos L.M., Calbo A.G., Sargent S.A. (2010). Climate changes and potential impacts on postharvest quality of fruit and vegetable crops: A review. Food Res. Int..

[47] Hartmann H.J., Kester D.E., Davies F.I., Geneve R.L. (2001). Plant Propagation: Principles and Practices.

[48] Balta M.F., Erol I.U., Özrenk K., Karakaya O., Uzun S. (2019). Investigation on propagation with softwood cuttings of Cornelian cherry (*Cornus mas* L.) genotypes. Türkiye Tarımsal Araştırmalar Derg..

[49] Costa J.M., Heuvelink E., van de Pol P. (2017). Propagation by Cuttings. the Reference Module in Life Sciences (Online).

[50] Pacholczak A., Jędrzejuk A., Sobczak M. (2017). Shading and natural rooting biostimulator enhance potential for vegetative propagation of dogwood plants (*Cornus alba* L.) via stem cuttings. S. Afr. J. Bot..

[51] Noor Camellia N.A., Thohirah L.A., Abdullah N.A.P., Mohd Khidir O. (2009). Improvement on Rooting Quality of *Jatropha curcas* using Indole Butyric Acid (IBA). Res. J. Agric. Biol. Sci..

[52] Guo X.F., Fu X.L., Zang D.K., Ma Y. (2009). Effect of auxin treatments, cuttings’ collection date and initial characteristics on *Paeonia* ‘Yang Fei Chu Yu’ cutting propagation. Sci. Hortic..

[53] Kumar R., Ahmed N., Sharma O.C., Lal S. (2014). Influence of auxins on rooting efficacy in carnation (*Dianthus caryophyllus* L.) cuttings. J. Hortic. Sci..

[54] Bijelić S.M., Gološin B.R., Cerović S.B., Bogdanović B.V. (2016). A comparison of grafting methods for the production of quality planting material of promising Cornelian cherry selections (*Cornus mas* L.) in Serbia. J. Agric. Sci. Technol..

[55] Cornescu F., Achim G., Cosmulescu S. (2020). Vegetative propagation of Cornelian cherry (*Cornus mas* L.) selections. Not. Sci. Biol..

[56] Strid A. (2016). Atlas of the Aegean Flora, Part 1: Text & Plates; Part 2: Maps; Englera, Volume 33.

[57] Madesis P., Ganopoulos I., Ralli P., Tsaftaris A. (2012). Barcoding the major Mediterranean leguminous crops by combining universal chloroplast and nuclear DNA sequence targets. Genet. Mol. Res..

[58] Chen S., Yao H., Han J., Liu C., Song J., Shi L., Zhu Y., Ma X., Gao T., Pang X. (2010). Validation of the ITS2 region as a novel DNA barcode for identifying medicinal plant species. PLoS ONE.

[59] Saitou N., Nei M. (1987). The neighbor-joining method: A new method for reconstructing phylogenetic trees. Mol. Biol. Evol..

[60] Felsenstein J. (1985). Confidence limits on phylogenies: An approach using the bootstrap. Evolution.

[61] Tamura K., Nei M., Kumar S. (2004). Prospects for inferring very large phylogenies by using the neighbor-joining method. Proc. Nat. Acad. Sci. USA.

[62] Kumar S., Stecher G., Li M., Knyaz C., Tamura K. (2018). MEGA X: Molecular evolutionary genetics analysis across computing platforms. Mol. Biol. Evol..

[63] Bryant P.H., Trueman S.J. (2015). Stem anatomy and adventitious root formation in cuttings of *Angophora*, *Corymbia* and *Eucalyptus*. Forests.

[64] Vavoura M.V., Badeka A.V., Kontakos S., Kontominas M.G. (2015). Characterization of four popular sweet cherry cultivars grown in Greece by volatile compound and physicochemical data analysis and sensory evaluation. Molecules.

[65] Roman I., Stanila A., Stanila S. (2013). Bioactive compounds and antioxidant activity of *Rosa canina* L. biotypes from spontaneous flora of Transylvania. Chem. Cent. J..

[66] Fattahi S., Jamei R., Hosseini Sarghein S. (2012). Antioxidant and antiradical activities of *Rosa canina* and *Rosa pimpinellifolia* fruits from West Azerbaijan. Iran. J. Plant Physiol..

[67] Lee H.S., Coates G.A. (1999). Vitamin C in frozen, fresh squeezed, unpasteurized, polyethylene-bottled orange juice: A storage study. Food Chem..

[68] Lambeth C.C. (1980). Juvenile-Mature correlations in Pinaceae and implications for early selection. For. Sci..

[69] Atay A.N. (2020). Deciphering morpho-agronomic determinants of the juvenile-mature phase change in apple progenies. Sci. Hortic..

